# Integrated palliative nursing interventions for older adults with cancer: Effects on quality of life, psychological outcomes, and symptom burden

**DOI:** 10.1017/S147895152610248X

**Published:** 2026-05-07

**Authors:** Ateya Megahed Ibrahim, Donia Elsaid Fathi Zaghamir

**Affiliations:** College of Nursing, Prince Sattam Bin Abdulaziz University, Alkharj, Saudi Arabia

**Keywords:** Palliative care, oncology nursing, quality of life, symptom management, psychological outcomes, older adults, ESAS, FACT-G, NCCN distress thermometer

## Abstract

**Objectives:**

Older adults with cancer frequently experience high symptom burden, psychological distress, and reduced quality of life. Integrating palliative nursing interventions into routine oncology care has the potential to improve these outcomes, yet evidence examining their measurable effects remains limited. This study aimed to examine the effects of integrated palliative nursing interventions on quality of life, psychological outcomes, and symptom burden among older adults with cancer.

**Methods:**

A quasi-experimental one-group pre-test–post-test design was conducted at King Khaled Hospital, Al-Kharj, Saudi Arabia, including 80 older adults (≥60 years) with confirmed cancer diagnosis. Participants received a structured 6-week integrated palliative nursing intervention comprising 12 sessions (2 sessions/week) addressing physical, psychological, social, functional, and spiritual needs. Outcome measures included the Functional Assessment of Cancer Therapy-General (FACT-G) for quality of life, the National Comprehensive Cancer Network (NCCN) Distress Thermometer for psychological outcomes, and the Edmonton Symptom Assessment System (ESAS-r) for symptom burden. Pre- and post-intervention assessments were conducted, and data were analyzed using paired *t*-tests, Pearson correlations, and multiple linear regression.

**Results:**

All 80 participants completed the study, and no attrition was observed during the 6-week intervention period. Post-intervention, participants demonstrated significant improvements in overall quality of life (FACT-G total: 39.65 ± 5.51 → 66.41 ± 6.25, *p* < .001) and all subscales. Distress scores (NCCN) decreased from 21.93 ± 2.49 to 6.99 ± 2.37 (*p* < .001), and total symptom burden (ESAS) declined from 63.56 ± 6.31 to 41.09 ± 6.88 (*p* < .001). Regression analysis identified baseline scores as significant predictors of post-intervention outcomes: pre-intervention FACT-G scores and cancer type for quality of life [*R*^2^ = 0.660, *F* (8, 71) = 17.199, *p* < .001), pre-intervention NCCN scores for distress (*R*^2^ = 0.219, F (8, 71) = 2.487, *p* = .019), and pre-intervention ESAS scores for symptom burden (*R*^2^ = 0.757, *F* (8, 71) = 27.697, *p* < .001). These results indicated that baseline status strongly predicts post-intervention outcomes, while demographic and clinical variables had minimal impact.

**Significance of the results:**

Structured integrated palliative nursing interventions significantly enhance quality of life and reduce psychological distress and symptom burden in older adults with cancer. Incorporating multidimensional, patient-centered palliative care within routine oncology practice can improve clinical outcomes, with baseline status serving as an important determinant of intervention effectiveness.

## Introduction

Cancer in older adulthood presents a distinct clinical and human challenge (Pawelec et al. 2026). As the global oncology population ages, a growing proportion of patients live not only with malignancy but also with frailty, multimorbidity, and reduced physiological reserve (Yadav et al. 2025). For many older adults, the cancer trajectory is characterized by fluctuating symptom intensity, treatment-related toxicities, psychological vulnerability, and progressive functional decline. In this context, survival alone is an insufficient marker of successful care (Locquet 2025; Lee et al. 2026). Quality of life, relief from suffering, preservation of dignity, and emotional stability become central priorities within oncology practice (Ahn et al. 2025; Paul 2026).

Palliative care has emerged as an essential component of comprehensive cancer management (Tarigan and Basabih 2026). Evidence consistently demonstrates that early integration of palliative care alongside disease-directed treatment improves symptom control, reduces psychological distress, enhances patient and family satisfaction, and may even positively influence survival (Lee et al. 2025). Within oncology, palliative care is no longer confined to end-of-life settings; rather, it represents a proactive, supportive approach that addresses pain, fatigue, dyspnea, anxiety, depression, existential concerns, and treatment burden across the illness continuum (Mejia and Blume 2026). However, despite this paradigm shift, implementation remains variable, and many older patients continue to experience unmanaged symptoms and unrecognized emotional distress (Mabonga et al. 2026).

Older adults with cancer are particularly vulnerable to complex symptom clusters (Wang et al. 2025; Hu et al. 2026). Pain frequently coexists with fatigue and sleep disturbances; dyspnea may be accompanied by anxiety; and functional limitations often contribute to depressive symptoms. These interconnected experiences amplify overall suffering and substantially diminish quality of life (Gonçalves et al. 2025). Furthermore, older patients may underreport symptoms, normalize distress as part of aging, or face barriers to accessing specialized supportive services. These realities underscore the importance of systematic assessment and integrated models of care within routine oncology settings (Sarac 2025).

Nursing practice occupies a central role in the operationalization of palliative care principles within oncology (El Fahli et al. 2025). Oncology nurses are uniquely positioned to conduct continuous symptom monitoring, provide psychosocial support, coordinate interdisciplinary referrals, educate patients and families, and facilitate shared decision-making (Turan 2026). When structured within an integrated palliative nursing framework, these roles can evolve from reactive symptom management toward proactive, multidimensional care delivery. Such integration ensures that physical symptoms, psychological wellbeing, and functional concerns are addressed simultaneously rather than in isolation (Shaheen and Sarwar 2025).

Despite the recognized importance of early palliative integration, evidence specifically evaluating structured, nurse-led palliative interventions among older adults with cancer remains limited (Singh et al. 2025). Most studies have focused either on specialist palliative consultations or on single-symptom management strategies, leaving a gap in understanding how comprehensive nursing-led interventions influence broader outcomes, such as quality of life, psychological wellbeing, and overall symptom burden. Generating rigorous evidence in this area is critical to advancing supportive oncology care models that are sustainable, scalable, and responsive to the needs of aging cancer populations.

Therefore, this study aims to examine the effects of integrated palliative nursing interventions on quality of life, psychological outcomes, and symptom burden among older adults with cancer. By situating palliative principles firmly within oncology nursing practice and evaluating their measurable impact, this research seeks to contribute to the growing movement toward holistic, person-centered cancer care that prioritizes comfort, dignity, and wellbeing alongside disease management.

## Method

### Study design

This study employed a quasi-experimental one-group pre-test–post-test design to evaluate the effects of integrated palliative nursing interventions on quality of life, psychological outcomes, and symptom burden among older adults with cancer. Participants were assessed at baseline (pre-intervention) and again following completion of the structured palliative nursing intervention program. This design was selected to examine within-subject changes over time in response to the intervention while maintaining feasibility within a real-world oncology care setting.

### Setting

The study was conducted at King Khaled Hospital, located in Al-Kharj, Saudi Arabia. The hospital is a governmental secondary-care facility that provides oncology and supportive services to patients in the Al-Kharj region and surrounding communities. Data collection took place in outpatient oncology clinics and relevant medical units where older adults with cancer receive follow-up care and symptom management. The setting provides a suitable clinical environment for implementing structured palliative nursing interventions integrated within routine oncology services.

### Sample size calculation

The sample size was calculated using G*Power version 3.1.9.7. For this quasi-experimental one-group pre-test–post-test design, a paired-samples *t*-test (two-tailed) was selected as the primary statistical test to detect differences between baseline and post-intervention scores.

**The following parameters were applied**:

- Effect size (Cohen’s *d*) = 0.50 (moderate effect)
- Significance level (α) = 0.05
- Statistical power (1 − β) = 0.80
- Allocation ratio = 1
- Two-tailed hypothesis

Based on these assumptions, the minimum required sample size was 34 participants. Considering the vulnerability of older adults with cancer and the potential for attrition due to disease progression, hospitalization, or withdrawal, an additional 25% was added to account for possible dropout. To enhance statistical robustness and ensure adequate power, the final target sample size was set at least 80 participants.

### Participants

Participants were recruited using a convenience sampling technique from eligible patients attending oncology outpatient services during the study period.

#### Inclusion criteria

Participants were eligible for inclusion if they:

1. Were aged 60 years or older.
2. Had a confirmed medical diagnosis of cancer (any type or stage).
3. Were receiving treatment or follow-up care at King Khaled Hospital.
4. Were clinically stable at the time of recruitment.
5. Were able to communicate verbally in Arabic or English.
6. Were cognitively capable of providing informed consent and completing study questionnaires.

#### Exclusion criteria

Participants were excluded if they:

1. Were diagnosed with severe cognitive impairment or advanced dementia.
2. Were in a critical condition requiring intensive care.
3. Had severe psychiatric disorders that could interfere with participation.
4. Were enrolled in another structured palliative intervention program during the study period.
5. Declined to provide informed consent.

### Data collection tools

#### Sociodemographic and clinical data questionnaire

A structured sociodemographic and clinical data questionnaire was developed by the researchers after reviewing relevant oncology and palliative care literature to collect baseline participant characteristics. The questionnaire was designed to obtain essential demographic variables, including age, gender, marital status, and educational level, as well as clinical characteristics such as cancer type, cancer stage, and duration since diagnosis. These variables were selected because of their documented association with quality of life, psychological distress, and symptom burden among oncology populations. The tool consisted of close-ended questions requiring categorical responses to facilitate statistical analysis.

### Quality of life assessment tool: functional assessment of cancer therapy – General

The Functional Assessment of Cancer Therapy- General (FACT-G) was developed by Cella and colleagues in 1993 as part of the FACIT measurement system to assess health-related quality of life in individuals with cancer (Cella et al. 1993). The instrument was designed specifically for oncology populations to provide a multidimensional evaluation of the impact of cancer and its treatment on patients’ wellbeing. The primary aim of FACT-G is to measure quality of life across physical, emotional, social/family, and functional domains, making it particularly suitable for intervention studies evaluating supportive and palliative care programs.

FACT-G (Version 4) consists of 27 items divided into 4 subscales: Physical Well-Being (7 items), Social/Family Well-Being (7 items), Emotional Well-Being (6 items), and Functional Well-Being (7 items). Patients respond based on how they have felt over the past 7 days using a 5-point Likert scale ranging from 0 (Not at all) to 4 (Very much). Several items are reverse-scored according to the scoring manual to ensure accurate domain calculation.

Scoring involves summing item responses within each subscale and then computing a total score. The total FACT-G score ranges from 0 to 108, with higher scores indicating better quality of life. Subscale scores can also be analyzed independently to examine specific domains of wellbeing. The tool takes approximately 5–10 minutes to complete and is suitable for older adults with cancer due to its clear wording and balanced structure.

Regarding psychometric properties, FACT-G demonstrates strong reliability and validity across cancer populations. Internal consistency reliability (Cronbach’s alpha) has been reported between 0.82 and 0.92 for the total scale, with subscale alphas typically above 0.70. Test–retest reliability coefficients exceed 0.85 in stable patients. Construct validity has been established through significant correlations with performance status measures and other quality-of-life instruments. FACT-G has been translated into multiple languages, including validated Arabic versions, and is widely used in oncology and palliative care research.

### Psychological outcomes assessment tool: NCCN distress thermometer

The NCCN Distress Thermometer (DT) was developed by the National Comprehensive Cancer Network (NCCN) as a rapid screening tool for psychological distress in oncology patients (NCCN 2016). The aim of the tool is to quickly identify patients experiencing significant emotional, social, or physical distress so that appropriate supportive interventions can be initiated. It is widely recommended in oncology and palliative care settings as a routine screening measure.

The Distress Thermometer consists of 2 main components: (1) a single-item visual analog scale resembling a thermometer ranging from 0 (No distress) to 10 (Extreme distress), where patients rate their overall distress over the past week, and (2) a Problem List that includes practical, family, emotional, spiritual/religious, and physical concerns. The Problem List helps clinicians identify the specific sources contributing to distress.

Scoring is straightforward: the patient circles a number from 0 to 10. A cut-off score of ≥ 4 is commonly used to indicate clinically significant distress requiring further assessment or intervention. The Problem List is analyzed descriptively to determine areas needing support. The tool requires only 2–5 minutes to complete and is highly feasible for older adults and medically fragile patients.

Psychometrically, the Distress Thermometer has demonstrated good validity and reliability in oncology populations. Sensitivity ranges from 0.65 to 0.80 and specificity from 0.70 to 0.80 when compared with structured psychiatric interviews or longer psychological scales such as the Hospital Anxiety and Depression Scale (HADS). Test–retest reliability coefficients have been reported around 0.80. Construct validity has been supported through moderate to strong correlations (*r* = 0.50–0.70) with standardized anxiety and depression measures. The tool has been translated and validated internationally, including Arabic oncology populations.

### Symptom burden assessment tool: Edmonton Symptom Assessment System

The Edmonton Symptom Assessment System (ESAS) was originally developed in 1991 by Bruera and colleagues at the Cross Cancer Institute in Edmonton, Canada, to provide a brief, clinically practical method for assessing symptom burden in patients receiving palliative care (Bruera et al. 1991). The revised version (ESAS-r) was later introduced to improve clarity of wording and standardization of symptom descriptors. The primary aim of ESAS-r is to quantitatively assess the severity of common symptoms experienced by patients with advanced cancer and those receiving palliative or supportive care, enabling clinicians and researchers to monitor symptom progression and evaluate response to interventions.

The ESAS-r consists of 9 core symptoms: pain, tiredness (fatigue), drowsiness, nausea, lack of appetite, shortness of breath, depression, anxiety, and overall wellbeing. Some versions also include sleep as an optional tenth item. Each symptom is rated on an 11-point numeric rating scale ranging from 0 to 10, where 0 indicates absence of the symptom and 10 represents the worst possible severity. The instrument is designed to assess symptoms over the past 24 hours, making it sensitive to short-term clinical changes and suitable for pre–post intervention studies.

Scoring involves analyzing individual symptom scores (0–10 each) and/or calculating a total symptom distress score (TSDS), obtained by summing all symptom ratings. With 9 symptoms, the total score ranges from 0 to 90; higher scores indicate greater overall symptom burden. Researchers may also compute sub-scores such as the physical symptom score (pain, fatigue, nausea, drowsiness, appetite, dyspnea) and psychological symptom score (depression and anxiety), depending on study objectives. The tool is simple, quick (takes approximately 3–5 minutes), and suitable for older adults with cancer.

Regarding psychometric properties, ESAS-r has demonstrated strong validity and reliability across oncology and palliative populations. Construct validity has been supported through significant correlations with other established quality-of-life and symptom measures. Internal consistency reliability has shown Cronbach’s alpha values ranging from approximately 0.79 to 0.89 in cancer samples. Test–retest reliability coefficients have been reported above 0.80 for stable patients. The tool is also highly responsive to clinical change, making it appropriate for intervention studies such as integrated palliative nursing programs. ESAS has been translated and validated in multiple languages, including Arabic versions used in Middle Eastern populations, with satisfactory reliability indices (α > 0.80).

#### Integrated palliative nursing intervention program

The study implemented a 6-week structured, nurse-led integrated palliative care program designed for older adults with cancer to improve quality of life, reduce psychological distress, and manage symptom burden. The program was delivered at King Khaled Hospital, Al-Kharj, Saudi Arabia, a governmental secondary-care facility providing comprehensive oncology and supportive services (Table 1). Notably, no attrition was observed during the study period, which may be attributed to the short intervention duration, inclusion of clinically stable participants, and the integration of the nurse-led intervention into routine care, enhancing adherence. This complete retention of participants strengthens the internal validity of the study and supports the reliability of the observed improvements in quality of life, psychological outcomes, and symptom burden.

**Table 1.** Session focus and activities

Week/Session	Focus Area	Objectives	Activities/Content	Duration
Week 1	Introduction & Assessment	Establish rapport, assess baseline quality of life, distress, and symptom burden	Orientation; baseline assessment using FACT-G, NCCN Distress Thermometer, ESAS; discuss patient expectations	60 min
Session 2	Understanding Cancer & Palliative Care	Educate on disease, symptom trajectory, and palliative principles	Interactive discussion and Q&A on cancer, treatment effects, and palliative care principles	60 min
Week 2	Physical Symptom Management – Part 1	Address pain, fatigue, sleep disturbances	Pain assessment, relaxation techniques, guided imagery, energy conservation strategies	90 min
Session 4	Physical Symptom Management – Part 2	Address dyspnea, appetite changes, nausea	Breathing exercises, dietary counseling, nausea reduction strategies	90 min
Week 3	Psychological Support – Part 1	Reduce anxiety and depressive symptoms	Cognitive-behavioral techniques, mindfulness, journaling, emotional expression	60−90 min
Session 6	Psychological Support – Part 2	Coping with uncertainty and loss	Stress management, problem-solving, family involvement	60−90 min
Week 4	Social & Family Well-Being	Strengthen support networks	Family counseling, communication skills training, social support enhancement	60−90 min
Session 8	Spiritual & Existential Care	Address spiritual/existential distress	Guided reflection, meaning-making exercises, hope enhancement	60 min
Week 5	Functional Well-Being & Rehabilitation	Maintain independence and functional capacity	Gentle exercise program, occupational therapy support, fall prevention strategies	60−90 min
Session 10	Integrative Symptom Management	Combine strategies for multi-symptom clusters	Personalized symptom management plan, reinforcement of self-care techniques	60−90 min
Week 6	Review & Empowerment	Consolidate learning and reinforce self-management	Reassessment using FACT-G, ESAS, NCCN Distress Thermometer, discuss progress and provide personalized care plan	60 min
Session 12	Closure & Follow-Up Planning	Ensure sustainability and continuity of care	Develop ongoing self-care plan, arrange referrals if needed, encourage family and community support	60 min

**Hospital Setting and Delivery Locations:**

- Outpatient Oncology Clinic: Main site for ambulatory patients, allowing structured group and individual sessions without disrupting routine treatment.
- Inpatient Oncology Units/Medical Wards: Bedside sessions for patients admitted for symptom management, chemotherapy-related side effects, or limited mobility.
- Day Care/Oncology Procedure Rooms: Quiet areas for private counseling, psychological support, or physical rehabilitation exercises.

**Program Structure:**

- Duration: 6 weeks
- Frequency: 2 sessions per week
- Total Sessions: 12
- Session Length: 60–90 minutes
- Delivery Methods:
  1. Face-to-face interactions led by trained oncology nurses
  2. Individual sessions for high-symptom or bedridden patients
  3. Small group sessions (4–6 participants) to encourage peer support and interactive learning
  4. Integration with routine oncology care to ensure feasibility and continuity.

**Key Components:**

- **Multidimensional care**: Addresses physical, psychological, social, functional, and spiritual domains simultaneously
- **Patient-centered and individualized**: Tailored to each patient’s symptom burden, functional status, and preferences
- **Family involvement**: Structured sessions to reinforce support networks and enhance adherence to self-care strategies
- **Regular assessment**: Continuous evaluation with validated tools (**FACT-G, NCCN Distress Thermometer, ESAS**) to monitor outcomes
- **Integration with oncology care**: Delivered alongside routine treatments to ensure feasibility, continuity, and patient accessibility

**Ethical Considerations:**

The study was conducted in accordance with the Declaration of Helsinki and approved by the Institutional Review Board of Prince Sattam bin Abdul-Aziz University. Written informed consent was obtained from all participants after explaining the study objectives, procedures, potential benefits, and risks. Participants were assured of voluntary participation, confidentiality, and the right to withdraw at any time without affecting their medical care. All personal and clinical data were anonymized and stored securely. The intervention was designed to minimize physical or psychological burden, with nurses continuously monitoring participants for any distress during sessions, ensuring ethical standards for vulnerable older adults with cancer were strictly upheld.

#### Statistical analysis

All analyses were conducted using SPSS version 26. Descriptive statistics (frequency, percentage, mean, and standard deviation) summarized participant characteristics. Paired *t*-tests evaluated pre–post differences in FACT-G, NCCN, and ESAS scores. Pearson correlation coefficients assessed relationships among quality-of-life, symptom burden, and distress. Multiple linear regression analyses identified significant predictors of post-intervention outcomes (FACT-G, NCCN distress, ESAS) while controlling for demographic and clinical variables. Significance was set at *p* < .05, with multiple comparisons highlighted at *p* < .001 where applicable.

## Results

Table 2 shows that the largest group aged 60–74 years (60%). Females constituted the majority (58.8%) compared to males (41.3%). Regarding marital status, single participants represented 30%, divorced 27.5%, widowed 25%, and married 17.5%. Education levels varied, with primary education accounting for 32.5%, secondary education 18.8%, university graduates 21.3%, and postgraduates 27.5%. Cancer types were varied, with leukemia (23.8%) and colorectal cancer (21.3%) being the most common, followed by lung (17.5%), breast (15%), liver (13.8%), and prostate (8.8%). Most participants were in Stage III (47.5%), followed by Stage II (25%) and Stage IV (27.5%). The majority had been diagnosed for more than 24 months (48.8%), with smaller proportions in 13–24 months (26.3%), 7–12 months (15%), and ≤ 6 months (10%).

**Table 2.** Sociodemographic and clinical characteristics of the study participants (*N* = 80)

Variable	Category	*n*	%
**Age group**	60−74 years	48	60
	≥75 years	32	40
**Gender**	Male	33	41.3
	Female	47	58.8
**Marital status**	Single	24	30.0
	Divorced	22	27.5
	Married	14	17.5
	Widowed	20	25.0
**Education level**	Primary education	26	32.5
	Secondary education	15	18.8
	University	17	21.3
	Postgraduate	22	27.5
**Cancer type**	Prostate	7	8.8
	Lung	14	17.5
	Leukemia	19	23.8
	Colorectal	17	21.3
	Liver	11	13.8
	Breast	12	15.0
**Cancer stage**	Stage II	20	25.0
	Stage III	38	47.5
	Stage IV	22	27.5
**Duration since diagnosis**	≤6 months	8	10.0
	7−12 months	12	15.0
	13−24 months	21	26.3
	>24 months	39	48.8

Table 3 demonstrates that Post-intervention scores for all FACT-G subscales significantly increased compared to pre-intervention values, indicating improved quality of life. Physical well-being improved from 10.89 ± 2.79 to 17.24 ± 2.91 (*p* < .001), social/family well-being from 10.46 ± 3.35 to 16.96 ± 3.45 (*p* < .001), emotional well-being from 9.01 ± 2.59 to 14.74 ± 3.03 (*p* < .001), and functional well-being from 9.29 ± 2.78 to 17.48 ± 3.48 (*p* < .001). The total FACT-G score increased from 39.65 ± 5.51 to 66.41 ± 6.25 (*p* < .001), indicating a substantial overall improvement in participants’ perceived quality of life after the intervention.

**Table 3.** Comparison of FACT-G subscales and total scores pre- and post-intervention (*N* = 80)

Domain	Pre (Mean ± SD)	Post (Mean ± SD)	Mean Difference	*t*	df	*p*-value
Physical Well-Being (GP)	10.89 ± 2.79	17.24 ± 2.91	−6.35 ± 1.86	−30.49	79	<.001***
Social/Family Well-Being (GS)	10.46 ± 3.35	16.96 ± 3.45	−6.50 ± 1.93	−30.14	79	<.001***
Emotional Well-Being (GE)	9.01 ± 2.59	14.74 ± 3.03	−5.73 ± 1.78	−28.79	79	<.001***
Functional Well-Being (GF)	9.29 ± 2.78	17.48 ± 3.48	−8.19 ± 1.98	−37.08	79	<.001***
**Total FACT-G Score**	**39.65 ± 5.51**	**66.41 ± 6.25**	**−26.76 ± 3.85**	**−62.19**	**79**	<.001*

* *p* < .05; *** *p* < .01; *** *p* < .001 (paired t-test). Values are presented as Mean ± Standard Deviation (SD). FACT-G = Functional Assessment of Cancer Therapy–General.

Table 4 reveals that all domains of the NCCN problem list and distress thermometer showed significant reductions post-intervention. Practical problems decreased from 3.55 ± 1.38 to 1.03 ± 0.93, family problems from 2.25 ± 0.96 to 0.79 ± 0.74, emotional problems from 3.56 ± 1.16 to 1.13 ± 0.77, spiritual scores from 0.61 ± 0.49 to 0.19 ± 0.39, and physical scores from 11.95 ± 2.26 to 3.86 ± 1.89 (all *p* < .001). Distress thermometer scores also decreased significantly from 21.93 ± 2.49 to 6.99 ± 2.37 (*p* < .001). Moderate pre–post correlations indicate consistency in participants’ response patterns across time.

**Table 4.** Comparison of NCCN problem and distress scores pre- and post-intervention (*N* = 80)

Domain	Pre (Mean ± SD)	Post (Mean ± SD)	Mean Difference ± SD	*t*	df	*p*-value	95% CI of difference	Pre–Post correlation (*r*)
Practical problems	3.55 ± 1.38	1.03 ± 0.93	2.53 ± 1.33	16.97	79	<0.001***	2.23–2.82	0.386, *p* < .001
Family problems	2.25 ± 0.96	0.79 ± 0.74	1.46 ± 0.89	14.78	79	<0.001***	1.27–1.66	0.484, *p* < .001
Emotional problems	3.56 ± 1.16	1.13 ± 0.77	2.44 ± 1.18	18.50	79	<0.001***	2.18–2.70	0.304, *p* = .006
Spiritual scores	0.61 ± 0.49	0.19 ± 0.39	0.43 ± 0.50	7.64	79	<0.001***	0.31–0.54	0.382, *p* < .001
Physical scores	11.95 ± 2.26	3.86 ± 1.89	8.09 ± 2.22	32.54	79	<0.001***	7.59–8.58	0.437, *p* < .001
**Distress (NCCN Thermometer)**	**21.93 ± 2.49**	**6.99 ± 2.37**	**14.94 ± 2.68**	**49.80**	**79**	<0.001***	**14.34–15.53**	**0.393, *p* < .001**

Table 5 shows that the total ESAS score decreased significantly from 63.56 ± 6.31 pre-intervention to 41.09 ± 6.88 post-intervention (*p* < .001), reflecting a substantial reduction in overall symptom burden. The high pre–post correlation (*r* = 0.853, *p* < .001) suggests strong reliability and consistency in symptom reporting.

**Table 5.** Comparison of total ESAS scores pre- and post-intervention (*N* = 80)

Domain	Pre (Mean ± SD)	Post (Mean ± SD)	Mean Difference ± SD	*t*	df	*p*-value	95% CI of difference	Pre–post correlation (r)
**Total ESAS Score**	63.56 ± 6.31	41.09 ± 6.88	22.48 ± 3.61	55.62	79	<0.001***	21.67–23.28	0.853, *p* < .001

Table 6 shows that the pre-intervention FACT-G scores negatively correlated with ESAS total scores (*r* = −0.138 to −0.144), while post-intervention correlations were weaker. Strong positive correlations were observed between pre and post FACT-G scores (*r* = 0.793**) and between pre and post ESAS scores (*r* = 0.853**). NCCN distress correlated positively with ESAS scores (*r* = 0.393**) and moderately with FACT-G pre-intervention (*r* = 0.255*), indicating that higher symptom burden and distress are associated with lower quality of life.

**Table 6.** Pearson correlations between quality-of-life, symptom burden, and distress scores (*N* = 80)

Variable	1	2	3	4	5	6
1. Total FACT-G Pre	1	0.793**	−0.138	−0.144	0.255*	0.134
2. Total FACT-G Post		1	−0.088	−0.081	0.210	0.187
3. Total ESAS Pre			1	0.853**	−0.231*	−0.122
4. Total ESAS Post				1	−0.229*	−0.083
5. NCCN Distress Pre					1	0.393**
6. NCCN Distress Post						1

Table 7 shows that multiple linear regression analysis identified pre-intervention FACT-G scores (β = 0.769, *p* < .001) and cancer type (β = −0.151, *p* = .041) as significant predictors of post-intervention quality of life. All other sociodemographic and clinical variables (age group, gender, marital status, education level, stage, and months since diagnosis) were not statistically significant. The overall regression model was statistically significant, *F* (8, 71) = 17.199, *p* < .001, explaining 66.0% of the variance in post-intervention quality-of-life scores (*R*^2^ = .660).

**Table 7.** Multiple linear regression predicting post-intervention quality of life (total FACT-G Post) (*N* = 80)

Predictor	B	SE	β	*t*	*P*
Constant	32.441	4.573	-	7.095	< .001
Total FACT-G Pre	0.873	0.085	0.769	10.291	< .001***
Age group	−0.508	0.919	−0.040	−0.553	.582
Gender	0.452	0.936	0.036	0.483	.631
Marital status	0.154	0.391	0.029	0.394	.695
Education level	0.125	0.380	0.024	0.330	.742
Cancer type	−0.621	0.298	−0.151	−2.084	.041*
Cancer stage	0.118	0.637	0.014	0.185	.854
Months since diagnosis	0.214	0.457	0.035	0.468	.641

*F*(8, 71) = 17.199.

*p* < .001.

*R*^2^ = .660.

Table 8 indicates that pre-intervention NCCN distress scores were the only significant predictor of post-intervention psychological distress (β = 0.361, *p* = .002). None of the demographic or clinical variables significantly predicted post-intervention distress levels. The regression model was statistically significant, *F* (8, 71) = 2.487, *p* = .019, accounting for 21.9% of the variance in post-intervention distress scores (*R*^2^ = .219).

**Table 8.** Multiple linear regression predicting post-intervention psychological distress (NCCN Distress Thermometer Post) (*N* = 80)

Predictor	B	SE	β	*t*	*p*
Constant	−3.206	2.940	-	−1.090	.279
NCCN Distress Thermometer (Pre)	.344	.107	.361	3.218	.002**
Age Group	.223	.528	.046	.423	.673
Gender	.866	.531	.181	1.631	.107
Marital status	.063	.234	.031	.269	.788
Education level	−.213	.215	−.108	−.990	.325
Cancer type	.061	.171	.039	.359	.721
Cancer stage	.084	.364	.026	.231	.818
Months since diagnosis	.302	.262	.129	1.151	.254

*F*(8, 71) = 2.487.

*p* = .019.

*R*^2^ = 0.219.

Table 9 demonstrates that pre-intervention ESAS scores were a strong and statistically significant predictor of post-intervention symptom burden (β = 0.849, *p* < .001). No demographic or clinical variables significantly contributed to the model. The regression model was highly significant, *F* (8, 71) = 27.697, *p* < .001, explaining 75.7% of the variance in post-intervention ESAS scores (*R*^2^ = .757).

**Table 9.** Multiple linear regression predicting post-intervention symptom burden (Total ESAS Post) (*N* = 80)

Predictor	B	SE	β	*t*	*p*
Constant	−19.450	5.473	-	−3.554	.001**
Total ESAS Pre	.926	.065	.849	14.231	< .001***
Age group	1.393	.846	.100	1.647	.104
Gender	.150	.853	.011	.176	.861
Marital status	.428	.363	.072	1.180	.242
Education level	−.062	.352	−.011	−.176	.861
Cancer type	−.266	.275	−.059	−.968	.336
Cancer stage	−.888	.589	−.094	−1.510	.136
Months since diagnosis	.434	.423	.064	1.026	.308

*F*(8, 71) = 27.69.

*p* < .001.

*R*^2^ = 0.757.

### Discussion

The total FACT-G score increased markedly from 39.65 ± 5.51 to 66.41 ± 6.25 (*p* < .001), with significant improvements observed in all 4 domains: physical, social/family, emotional, and functional well-being. From the researcher’s perspective, this substantial increase reflects the multidimensional design of the intervention. Unlike fragmented symptom-focused approaches, the integrated program simultaneously targeted pain control, psychological coping, family communication, spiritual reflection, and functional independence. The largest mean improvement was observed in functional well-being, suggesting that empowering patients with self-management strategies and rehabilitation exercises enhanced their sense of autonomy and daily role performance.

Regression analysis further confirmed these findings. Table 7 shows that pre-intervention FACT-G scores (β = 0.769, *p* < .001) and cancer type (β = − 0.151, *p* = .041) were significant predictors of post-intervention quality of life. The other demographic and clinical variables (age group, gender, marital status, education level, stage, and months since diagnosis) were not significant. The model explained 66.0% of the variance in post-intervention quality-of-life scores (*R*^2^ = 0.660, *F* (8, 71) = 17.199, *p* < .001), indicating that while baseline quality of life is the dominant predictor, meaningful improvements occurred across diverse patient groups.

These findings are further reinforced by recent high-quality evidence. For example, Haroen et al. (2025) reported in BMC Palliative Care that early palliative care significantly improves psychological well-being, functional status, and health-related quality of life among cancer patients and their caregivers. Similarly, Getie et al. (2025), in a global systematic review and meta-analysis published in PLOS ONE, demonstrated that integrated palliative care yields superior quality-of-life outcomes compared with standard oncology care. In addition, Aghahosseini et al. (2025) showed, through a quasi-experimental study, that palliative care interventions significantly enhanced patients’ quality of life and satisfaction with care.

These contemporary findings align with the original validation study of the FACT-G by Cella et al. (1993), which confirmed the instrument’s sensitivity to clinical change in oncology populations. Moreover, the landmark randomized controlled trial by Temel et al. (2010), published in the *New England Journal of Medicine*, and demonstrated that early palliative care significantly improved quality of life among patients with metastatic lung cancer. Likewise, Zimmermann et al. (2014) found that early palliative care enhanced overall quality of life and satisfaction with care compared to standard oncology treatment. Collectively, these foundational and recent studies consistently support the present findings, confirming that integrating palliative care principles within routine oncology services leads to meaningful and clinically significant improvements in patients’ perceived well-being, functional outcomes, and overall quality of life.

In the same vein, psychological distress decreased significantly post-intervention, with distress thermometer scores dropping from 21.93 ± 2.49 to 6.99 ± 2.37 (*p* < .001). Significant reductions were also observed across practical, emotional, family, spiritual, and physical problem domains. From the researcher’s viewpoint, this decline can be attributed to structured psychological support sessions incorporating cognitive-behavioral techniques, mindfulness strategies, family involvement, and spiritual counseling. The intervention directly addressed emotional expression and coping with uncertainty, which are central contributors to distress in oncology populations.

Regression analysis (Table 8) revealed that pre-intervention NCCN distress scores (β = 0.361, *p* = .002) were the only significant predictor of post-intervention distress, explaining 21.9% of the variance (*R*^2^ = 0.219, *F* (8, 71) = 2.487, *p* = .019). Other demographic and clinical factors were not significant. Clinically, this suggests that patients with higher initial distress remain more vulnerable, highlighting the need for early identification and potentially intensified psychological support for high-risk individuals.

These results are consistent with the recommendations of the NCCN, which identifies distress as the “sixth vital sign” in oncology and strongly advocates for routine distress screening and early supportive care interventions as standards of comprehensive cancer management. The NCCN Distress Management Guidelines emphasize systematic assessment, timely referral, and multidisciplinary psychosocial support to optimize patient outcomes (Waller et al. 2012; Lazenby 2014; NCCN 2016). Empirical evidence further supports this approach: structured distress screening combined with personalized triage significantly reduces distress, anxiety, and depressive symptoms among cancer patients (Carlson et al. 2012). Similarly, comprehensive psychosocial oncology programs have been shown to effectively decrease emotional burden and improve psychosocial well-being (Carlson and Bultz 2003).

In addition, recent evidence by Wang and Ding (2025), published in BMC Palliative Care, showed that integrated palliative care significantly improves overall quality of life among patients with advanced cancer, although its direct effects on anxiety and depression were limited. Together, these findings suggest that while integrated palliative care enhances global quality of life, optimal psychosocial outcomes are achieved when combined with structured distress screening and targeted psychological interventions in line with NCCN recommendations.

On the other hand, Bader et al. (2025), published in the *American Journal of Hospice and Palliative Medicine*, indicated that although integrating palliative care improves overall quality of life in patients with advanced cancer, its effect on psychological status particularly depression and anxiety was limited and statistically non-significant. This highlights the need for additional structured psychosocial interventions to achieve meaningful improvements in emotional outcomes.

Regarding symptom burden, the total ESAS score significantly decreased from 63.56 ± 6.31 to 41.09 ± 6.88 (*p* < .001), reflecting a substantial reduction in symptom burden. From the researcher’s interpretation, this improvement is likely attributable to systematic symptom assessment, individualized care planning, and continuous monitoring throughout the 6-week program. Physical symptom domains such as pain, fatigue, dyspnea, and nausea were directly addressed through education, breathing exercises, dietary guidance, and relaxation techniques.

Regression analysis (Table 9) confirmed that pre-intervention ESAS scores (β = 0.849, *p* < .001) were the strongest predictor of post-intervention symptom burden, explaining 75.7% of the variance (*R*^2^ = 0.757, *F* (8, 71) = 27.697, *p* < .001). Other demographic and clinical variables did not significantly contribute. This suggests that while the intervention was effective across the cohort, patients with severe baseline symptoms may require longer or more intensive support.

The ESAS was originally developed by Bruera et al. (1991) to enhance symptom monitoring in palliative populations, and subsequent research has consistently confirmed its responsiveness to clinical interventions. Hui and Bruera (2017) emphasized that early integration of palliative care into oncology services significantly improves symptom control. Additionally, a systematic review by Haun et al. (2017) concluded that palliative care interventions are associated with reduced symptom burden and improved quality of life in adult cancer patients.

These findings are further supported by recent quasi-experimental studies conducted in Egypt. For instance, Ibrahim et al. (2024a) demonstrated that a comprehensive rehabilitation palliative care program significantly enhanced quality of life for patients with terminal cancer and their informal caregivers. Similarly, Ibrahim et al. (2024b) reported that a structured palliative care education program for caregivers of cancer patients undergoing chemotherapy improved caregivers’ competence and patients’ overall well-being. Furthermore, Ibrahim et al. (2025) showed that palliative care interventions play a critical role in symptom management for patients with chronic kidney disease, confirming the applicability of these approaches beyond oncology. Collectively, these studies corroborate that systematic palliative care interventions whether focused on patients or caregivers effectively improve symptom control, reduce burden, and enhance overall quality of life, in line with the responsiveness of the ESAS and previous international evidence.

Furthermore, correlation analysis demonstrated significant relationships among study variables. Higher symptom burden and distress were associated with lower quality of life, consistent with the theoretical foundation of holistic palliative care. Pre-intervention FACT-G scores negatively correlated with ESAS scores, confirming that symptom severity directly compromises overall well-being. Additionally, NCCN distress correlated positively with ESAS scores (*r* = 0.393, *p* < .001), indicating that unmanaged physical symptoms exacerbate emotional distress.

These findings align with Ferrell et al.’s (2017) model of palliative nursing care, which emphasizes the interconnected nature of physical, psychological, social, and spiritual domains. They also support the biopsychosocial framework underpinning modern palliative oncology practice.

Interestingly, most demographic variables (age, gender, marital status, education, cancer stage, and duration since diagnosis) did not significantly predict post-intervention outcomes. Only cancer type showed a modest effect on post-intervention quality of life (β = −0.152, *p* = .040). From the researcher’s perspective, this suggests that the integrated intervention was broadly effective across diverse patient groups, reinforcing its feasibility and applicability in heterogeneous oncology populations.

This finding is consistent with previous research indicating that early palliative care benefits patients regardless of demographic background (Zimmermann et al. 2014; Haun et al. 2017). It further supports the universality of palliative nursing principles when systematically applied.

Overall, the findings strongly support the effectiveness of integrating structured palliative nursing interventions within routine oncology care. The magnitude of improvement in quality of life, combined with significant reductions in distress and symptom burden, indicates both statistical and clinical significance. The high *R*^2^ values in regression models for quality of life (0.658) and symptom burden (0.748) demonstrate that baseline status plays a dominant role, but meaningful improvements are achievable through structured nursing interventions. From a clinical perspective, the results advocate for routine implementation of nurse-led integrated palliative programs in outpatient and inpatient oncology settings. Embedding systematic assessment tools such as FACT-G, NCCN Distress Thermometer, and ESAS within regular care pathways enhances early identification of patient needs and ensures timely intervention.

## Conclusion

In summary, the evidence consistently demonstrates that integrating palliative care into routine oncology and chronic disease management improves overall quality of life, enhances symptom control, and supports both patients and informal caregivers. While global quality-of-life outcomes show significant improvement, psychological outcomes such as anxiety and depression may require additional structured interventions. Early incorporation of palliative principles, combined with systematic distress screening and caregiver education, maximizes the benefits of supportive care across multiple patient populations.

### Recommendation

It is recommended that healthcare systems adopt comprehensive palliative care programs that integrate symptom monitoring tools (e.g., ESAS), structured psychosocial support, and caregiver education as standard practice for patients with advanced cancer and other chronic life-limiting conditions. Policymakers and institutions should prioritize training healthcare professionals in these approaches and ensure systematic distress screening is embedded in routine clinical workflows to optimize patient and caregiver outcomes.

### Limitations

Despite the demonstrated benefits, the current evidence has some limitations. Many studies were conducted in single centers or specific regions, which may limit generalizability. Additionally, variations in intervention content, duration, and delivery methods make direct comparisons challenging, and some psychological outcomes remain underexplored. Future multi-center, standardized trials are needed to further clarify the impact of integrated palliative care on emotional and psychosocial outcomes.

## Data Availability

The original contributions presented in this study are available within the manuscript. For additional inquiries, please contact the corresponding author.
